# The Gut Microbiota Protects Bees from Invasion by a Bacterial Pathogen

**DOI:** 10.1128/Spectrum.00394-21

**Published:** 2021-09-15

**Authors:** Margaret I. Steele, Erick V. S. Motta, Tejashwini Gattu, Daniel Martinez, Nancy A. Moran

**Affiliations:** a Department of Integrative Biology, University of Texas at Austin, Austin, Texas, USA; University of Texas at San Antonio

**Keywords:** *Apis mellifera*, *Serratia marcescens*, T6SS, colonization resistance, microbiota

## Abstract

Commensal microbes in animal guts often help to exclude bacterial pathogens. In honey bees, perturbing or depleting the gut microbiota increases host mortality rates upon challenge with the opportunistic pathogen Serratia marcescens, suggesting antagonism between S. marcescens and one or more members of the bee gut microbiota. In laboratory culture, S. marcescens uses a type VI secretion system (T6SS) to kill bacterial competitors, but the role of this T6SS within hosts is unknown. Using infection assays, we determined how the microbiota impacts the abundance and persistence of S. marcescens in the gut and visualized colocalization of S. marcescens with specific community members *in situ*. Using T6SS-deficient S. marcescens strains, we measured T6SS-dependent killing of gut isolates *in vitro* and compared the persistence of mutant and wild-type strains in the gut. We found that S. marcescens is rapidly eliminated in the presence of the microbiota but persists in microbiota-free guts. Protection is reduced in monocolonized and antibiotic-treated bees, possibly because different symbionts occupy distinct niches. Serratia marcescens uses a T6SS to antagonize Escherichia coli and other S. marcescens strains but shows limited ability to kill bee symbionts. Furthermore, wild-type and T6SS-deficient S. marcescens strains achieved similar abundance and persistence in bee guts. Thus, an intact gut microbiota offers robust protection against this common pathogen, whose T6SSs do not confer the ability to compete with commensal species.

**IMPORTANCE** Bacteria living within guts of animals can provide protection against infection by pathogens. Some pathogens have been shown to use a molecular weapon known as a T6SS to kill beneficial bacteria during invasion of the mouse gut. In this study, we examined how bacteria native to the honey bee gut work together to exclude the opportunistic pathogen Serratia marcescens. Although S. marcescens has a T6SS that can kill bacteria, bee gut bacteria seem resistant to its effects. This limitation may partially explain why ingestion of S. marcescens is rarely lethal to insects with healthy gut communities.

## INTRODUCTION

Serratia marcescens is an environmental bacterium that frequently acts as an opportunistic pathogen of insects (1, 2) and as a nosocomial pathogen of humans (3). In insects, S. marcescens is typically lethal only when injected into the hemolymph, but in some hosts (4–6) it can infect orally, escape from the gut into the body cavity, and ultimately kill the host. S. marcescens likely interacts with the native gut microbiota as it passes through the gut, suggesting that competition with commensal bacteria could modulate pathogenicity.

Several S. marcescens strains were recently identified as opportunistic pathogens of honey bees (6). These strains are capable of causing lethal infections when introduced orally, with greater mortality rates among bees whose gut microbiota has been perturbed by exposure to antibiotics or agrochemicals (7, 8). In honey bees, 10 core taxa comprise >95% of the gut microbiota (9–12). This community contributes to host health by promoting weight gain (13), assisting in breakdown of otherwise indigestible carbohydrates (14–16), and conferring colonization resistance to certain pathogens (17). Symbiont biofilms line regions of the gut (18) and may act as a barrier against pathogen invasion. Also, several symbionts contain type VI secretion systems (T6SSs) (19), which may be used to kill potential competitors, including pathogens. However, some pathogens may overcome these defenses by producing a chemical arsenal to kill gut bacteria and disrupt the microbiota. For example, T6SSs are increasingly recognized as important mediators of antagonism between Gram-negative bacteria (20). These protein complexes, which are used to deliver a variety of toxins through membranes of adjacent cells, are common in Gram-negative bacteria (20–22). T6SS-mediated killing of bacterial competitors has been demonstrated *in vitro* in a variety of species (23–27), including S. marcescens Db11, a close relative of S. marcescens strains isolated from honey bees (24, 28, 29). It is not known whether S. marcescens uses a T6SS to compete with commensal bacteria during infection of hosts. However, T6SSs have been shown to mediate interactions between commensal gut bacteria and other pathogens in mice (30–32) and fruit flies (33), suggesting that T6SSs may be a viable mechanism for opportunistic pathogens to overcome colonization resistance provided by native microbiotas.

In this study, we examined the effects of the microbiota on S. marcescens persistence after oral exposure. We observed rapid elimination of S. marcescens from bees previously colonized by gut symbionts and competition for space between S. marcescens and commensals in the ileum. We also tested the hypothesis that this common opportunistic pathogen, which has a well-studied antibacterial T6SS (24, 28, 29), would antagonize gut commensals during infection. When grown in culture, S. marcescens does use a T6SS to antagonize Escherichia coli and other S. marcescens strains, as well as the bee symbiont *Gilliamella*. However, inactivating the T6SS did not alter S. marcescens success in the gut, under any conditions tested. Overall, our results indicate that multiple symbiont species contribute to colonization resistance toward an opportunistic pathogen. Furthermore, the T6SSs of this pathogen do not enable it to overcome this community-conferred resistance and are instead likely to be deployed in some other ecological context.

## RESULTS

### The microbiota drives elimination of S. marcescens from the bee gut.

**FIG 1:**
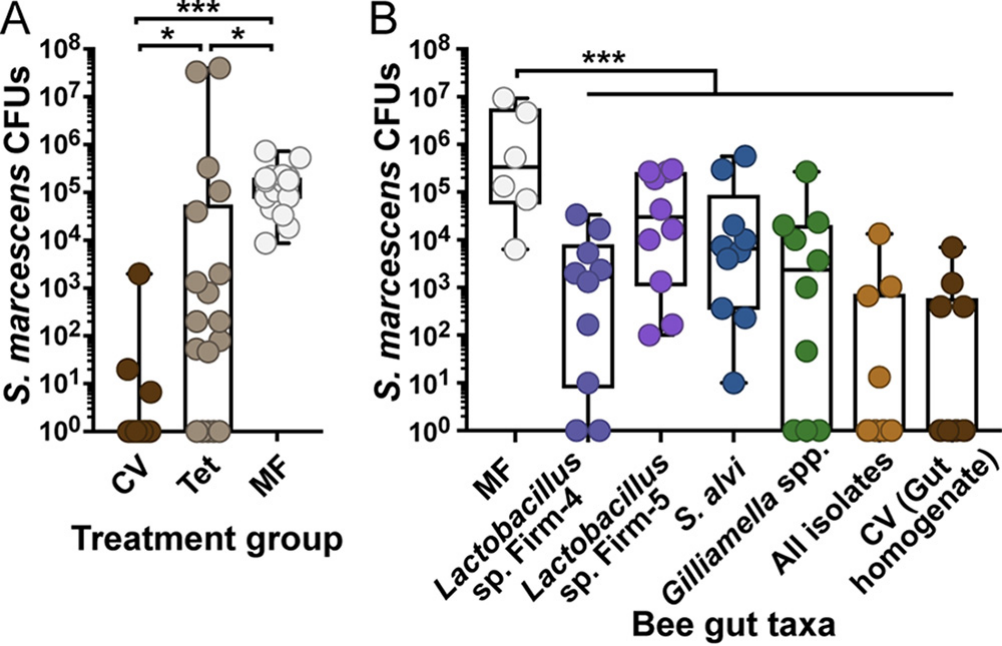
Serratia marcescens is eliminated from the guts of bees colonized by commensal species. (A) Total S. marcescens kz11 abundance in the midgut and hindgut of bees with a conventional, perturbed, or absent gut microbiota. Newly emerged bees from a single hive were inoculated with a conventional gut community (CV), inoculated with a conventional community and later treated with Tet (Tet), or kept microbiota free (MF). *, *P < *0.05; ***, *P < *0.005, Kruskal-Wallis test with Dunn’s multiple-comparison test. (B) S. marcescens abundance in bees colonized by individual gut taxa. MF bees were inoculated with representative strains of core gut taxa (*Lactobacillus* sp. Firm-4 DSM 26254 and DSM 26255, *Lactobacillus* sp. Firm-5 wkB8 and wkB10, S. alvi wkB2, G. apicola wkB1 and PEB0154, and G. apis PEB0162 and PEB0183), all isolates in combination, or homogenized gut of a bee collected from the hive. Bees were exposed for 1 day to 4 × 10^8^ S. marcescens cells/ml in sugar syrup and dissected 1 day after the end of exposure. S. marcescens abundance was quantified by counting CFU. Box and whisker plots show the minimum, first quartile, median, third quartile, and maximum. ***, *P < *0.005, one-way ANOVA with Tukey’s multiple-comparison test.

Bees with a perturbed gut microbiota have reduced survival rates after challenge with S. marcescens, compared to bees with a healthy microbiota (7, 8), but whether the microbiota contributes to host survival by killing S. marcescens is unknown. To determine whether S. marcescens abundance is reduced in bees with a healthy microbiota, we exposed bees with a conventional microbiota (CV), CV bees treated with tetracycline (Tet), or microbiota-free (MF) bees to S. marcescens kz11, a pathogenic isolate from bees (6, 8). Tet treatment was included to account for differences in metabolism, weight gain, and immune development between MF and CV bees (13, 34). Treatment with Tet reduces the abundance of gut bacteria and increases susceptibility to lethal infections but does not eliminate the commensal microbiota (8). One day after exposure, 100% of MF bees were infected, with an average of 1.85 × 10^5^ S. marcescens CFU per gut, while 83.3% of CV bees contained no living S. marcescens cells (Fig. 1A). Bees in the Tet treatment group exhibited an intermediate and highly variable phenotype, with 4.10 × 10^6^ S. marcescens CFU per gut on average and elimination of S. marcescens in 27.8% of bees. Loss of colonization resistance in Tet bees suggests that the gut microbiota may directly contribute to exclusion of S. marcescens from the gut, rather than acting through an indirect mechanism such as priming of the host immune system (6). Sucrose consumption is reduced in MF bees relative to CV bees (13), but differences in feeding behavior do not affect S. marcescens levels, as CV, Tet, MF, and MF Tet bees (MF bees treated with Tet) contained similar numbers of S. marcescens cells immediately after exposure (see Fig. S1A in the supplemental material), with differences between CV bees and other treatment groups developing over the next 24 h (see Fig. S1B and C). Additionally, protection from S. marcescens may vary without perturbation, due to variation in strains and relative abundances of gut taxa or due to host conditions. We observed a 1,000-fold difference in average S. marcescens CFU per gut between age-controlled bees from the same hive inoculated with conventional communities from two different hives (see Fig. S2).

### Multiple species contribute to elimination of S. marcescens.

Increased persistence of S. marcescens in Tet-treated bees suggests that microbiota composition is important for colonization resistance. To determine whether individual symbionts are capable of suppressing S. marcescens, we inoculated MF bees with representative isolates of the four most abundant core gut taxa, namely, *Lactobacillus* Firm-4 (DSM 26254 and DSM 26255), *Lactobacillus* Firm-5 (wkB8 and wkB10), Snodgrassella alvi (wkB2), or *Gilliamella* spp. (wkB1, PEB0154, PEB0162, and PEB0183). We also inoculated bees with all 9 isolates in combination or with homogenized hindgut from a hive bee (CV). After 5 days, the time required for microbiota establishment (18), bees were fed sucrose syrup containing S. marcescens at an optical density (OD600) of 0.5 for 1 day and then returned to a diet of sterile sucrose solution. Serratia marcescens abundance was quantified 1 day later. Serratia marcescens CFU were reduced in bees inoculated with any of the core gut taxa, relative to MF bees (*P* < 0.005, one-way analysis of variance [ANOVA] with Tukey’s multiple-comparison test) (Fig. 1B). MF bees were infected with an average 2.5 × 10^6^ S. marcescens CFU per gut, in contrast to bees inoculated with Firm-4, Firm-5, S. alvi, or *Gilliamella* spp., which contained an average of 6.1 × 10^3^, 1.1 × 10^5^, 9.1 × 10^4^, and 3.3 × 10^4^ S. marcescens CFU per gut, respectively. The combined effect of these four taxa exceeded that of any taxon alone, with 1.5 × 10^3^ S. marcescens CFU per gut and only 40% of bees still infected. These numbers resemble those in bees inoculated with gut homogenate (40% infected, with 9.0 × 10^2^ CFU per gut). Therefore, antagonism of S. marcescens by the bee gut microbiota is likely to be a combined effect of multiple species.

### Commensal bacteria may exclude S. marcescens from regions of the gut.

**FIG 2:**
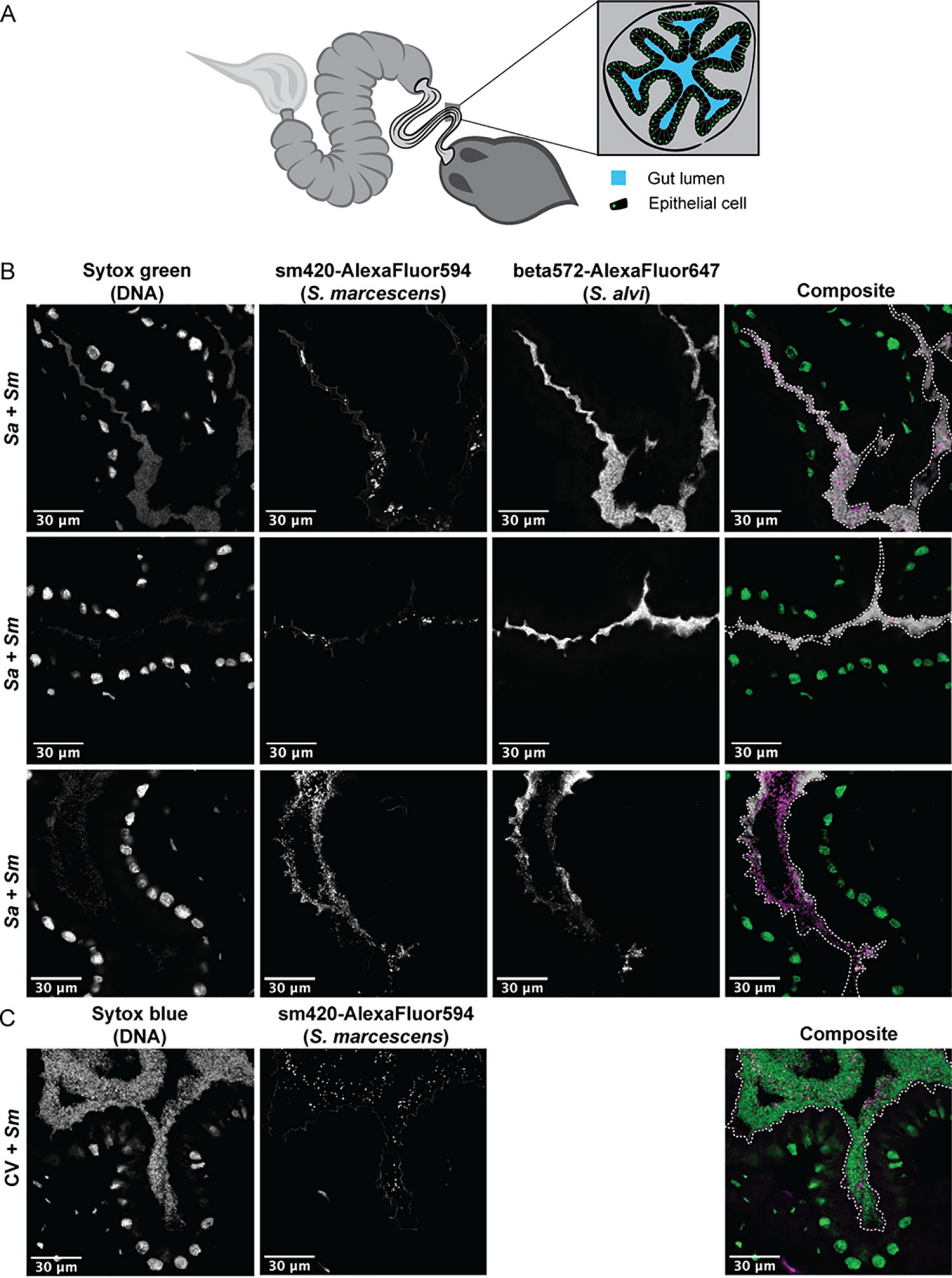
Gut commensals compete with Serratia marcescens for space. (A) Diagram of the honey bee gut, showing a cross-section of the ileum. (B) Representative cross-sections of ilea from bees colonized by S. alvi (white) and S. marcescens (magenta). MF bees were inoculated with S. alvi wkB2 and exposed to S. marcescens after 5 days. S. alvi and S. marcescens were visualized using fluorescent probes that hybridize to 16S rRNA, while Sytox green stain indicates the presence of host nuclei and bacterial cells (green). (C) Cross-section of the ileum of a bee inoculated with gut homogenate (CV) and then exposed to S. marcescens (magenta). Sytox blue stain was used to label host nuclei and bacterial cells (green). Dashed white lines on the composite images outline the lumen of the gut.

To determine whether S. marcescens physically interacts with gut symbionts, we used fluorescence *in situ* hybridization (FISH) microscopy to visualize the location of S. marcescens within the ilea of CV bees and bees monocolonized by S. alvi. The ileum is bordered by multiple folds of the epithelial layer, creating narrow grooves that are usually filled with gut symbionts (Fig. 2A). S. alvi colonizes the surface of the host epithelium and fills the lumen only in narrow regions of the ileum (18, 35). In MF bees, S. marcescens colonizes the same niche (see Fig. S3). When bees monocolonized by S. alvi are infected with S. marcescens, the pathogen infiltrates this space, forming small clusters of cells that are frequently separated from the host epithelium by a layer of symbiotic bacteria (Fig. 2B). Occasionally, larger populations of S. marcescens cells colonize the ilea of monoinoculated bees, suggesting that S. marcescens displaces S. alvi or exploits niches left open by the absence of other members of the microbiota (Fig. 2; also see Fig. S3). In CV bees 1 day after exposure, S. marcescens cells are scattered throughout a dense layer of commensal bacteria but do not seem to form aggregates, as in monocolonized bees (Fig. 2C). Thus, a diverse gut community appears capable of more fully exploiting niches within the gut, allowing greater exclusion of S. marcescens.

### Serratia marcescens kz11 contains two T6SSs.

**FIG 3:**
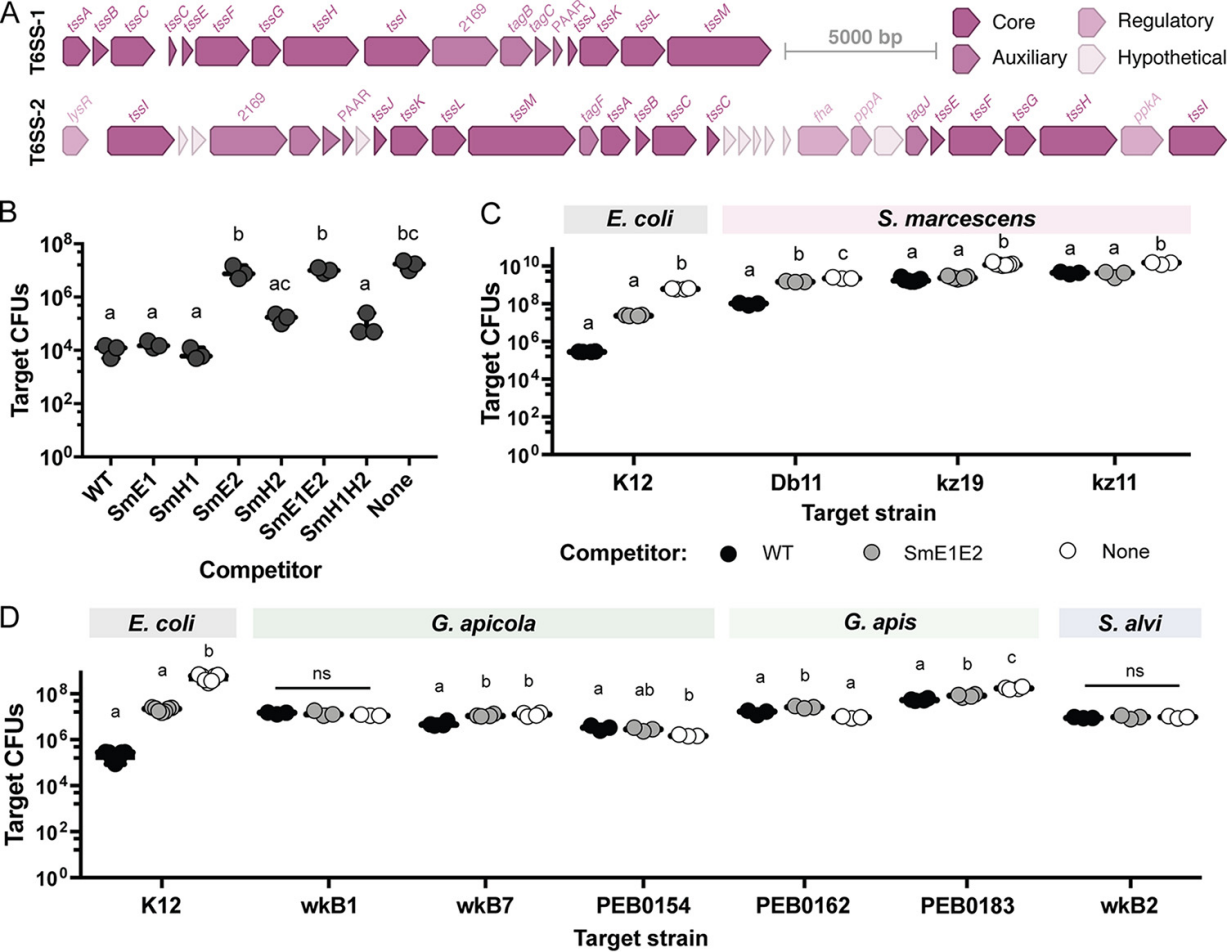
Serratia marcescens kz11 uses a T6SS to antagonize other S. marcescens strains and one bee commensal. (A) Open reading frame (ORF) map of the T6SS loci in S. marcescens kz11, with structural genes in dark pink, auxiliary genes (such as adapters and effectors) in medium pink, genes for posttranscriptional regulation in light pink, and hypothetical genes encoding proteins of unknown function in white. (B) Recovery of E. coli K-12 Tn*7*-Gm^R^ after 4 h of coculture with the indicated S. marcescens strains. Competitions began with approximately 10^7^ E. coli cells and a 1:4 ratio of E. coli to S. marcescens. (C) Recovery of E. coli K-12 and S. marcescens strain Db11, kz19, and kz11 CFU after 4 h of coculture with WT S. marcescens kz11 (black), SmE1E2 (gray), or buffer (white). (D) Recovery of E. coli K-12 and gut commensal G. apicola wkB1, wkB7, and PEB0154, G. apis PEB0162 and PEB0183, and S. alvi wkB2 CFU after coculture with S. marcescens. Target CFU were measured through plate counts on selective media. Letters indicate significant differences between treatment groups (one-way ANOVA with Tukey’s multiple-comparison test, *P < *0.05). ns, not significant.

Serratia marcescens kz11 contains two complete sets of genes, each encoding the 13 required structural components of T6SSs, which are grouped into two loci (Fig. 3A). These loci share ≥90% nucleotide identity (≥70% coverage) with T6SS loci present in other S. marcescens strains. Both T6SSs are widespread within the genus, but T6SS-2 (which is also found in S. marcescens Db11) is more common among sequenced genomes (492 of 670 genomes) than T6SS-1 (189 of 670 genomes). We constructed in-frame replacements of genes encoding required structural components (*tssE* or *tssH*) of the two T6SSs (SmE1, SmH1, SmE2, and SmH2), as well as double replacements (SmE1E2 and SmH1H2). Strains lacking structural components of T6SS-2 had reduced ability to antagonize E. coli K-12 during *in vitro* competition assays, with deletion of *tssE2* having a greater effect (Fig. 3B). In contrast, deletion of T6SS-1 genes had no effect on T6SS-mediated killing, indicating that T6SS-1 is not used to target E. coli or is not active under the tested conditions.

### Serratia marcescens T6SSs have a limited target range.

To determine whether kz11 uses a T6SS to kill other S. marcescens strains, we set up pairwise competitions *in vitro* with a starting ratio of 10:1 attacker (wild-type [WT] or T6SS-deficient S. marcescens kz11) to target (S. marcescens Db11, kz19, or rifampin [Rif]-resistant kz11) (Fig. 3C). Fewer CFU were recovered from competitions than from competitor-free (phosphate-buffered saline [PBS]) controls for all three strains. More Db11 CFU were recovered after coculture with T6SS-SmE1E2 than with WT S. marcescens. While there was no significant difference in the number of kz19 CFU after coculture with either competitor, kz19 represented a larger percentage of the CFU recovered from coculture with SmE1E2 (see Fig. S4A). As expected, there was no difference in the number of WT kz11 CFU recovered after coculture with the kz11-derived mutants. As for E. coli K-12, antagonism of Db11 and kz19 appeared dependent on T6SS-2, but not T6SS-1, under the tested conditions (see Fig. S4B). Additionally, S. marcescens strains vary in susceptibility to T6SS-2, with Db11, an isolate from *Drosophila*, being more susceptible than kz19, an isolate from honey bees (8), to antagonism by kz11.

We tested the ability of S. marcescens to antagonize *Gilliamella* spp. and S. alvi, the two most abundant Gram-negative taxa in the honey bee gut. We recovered more Gilliamella apicola wkB7, Gilliamella apis PEB0162, and Gilliamella apis PEB0183 CFU after coculture with S. marcescens SmE1E2, relative to coculture with the WT strain, but found no difference in recovery of S. alvi wkB2, G. apicola wkB1, and G. apicola PEB0154 CFU (Fig. 3C). When fewer target CFU were recovered after coculture with WT S. marcescens, the target strain also represented a smaller proportion of the total recovered CFU (see Fig. S4). However, while statistically significant, the effect of the S. marcescens T6SSs on *Gilliamella* spp. *in vitro* was less than the effect on E. coli K-12 and S. marcescens Db11. Furthermore, there was no difference in *Gilliamella* or S. alvi abundance, based on 16S rRNA gene copies, for CV bees infected with WT S. marcescens versus SmE1E2 (see Fig. S5), suggesting that S. marcescens T6SSs do not affect the abundance of these species *in vivo*. We also found no difference in *Gilliamella* or *S. alvi* abundance between bees in which S. marcescens had been eliminated and bees in which it persisted.

### Serratia marcescens T6SSs do not affect fitness in the bee gut.

**FIG 4:**
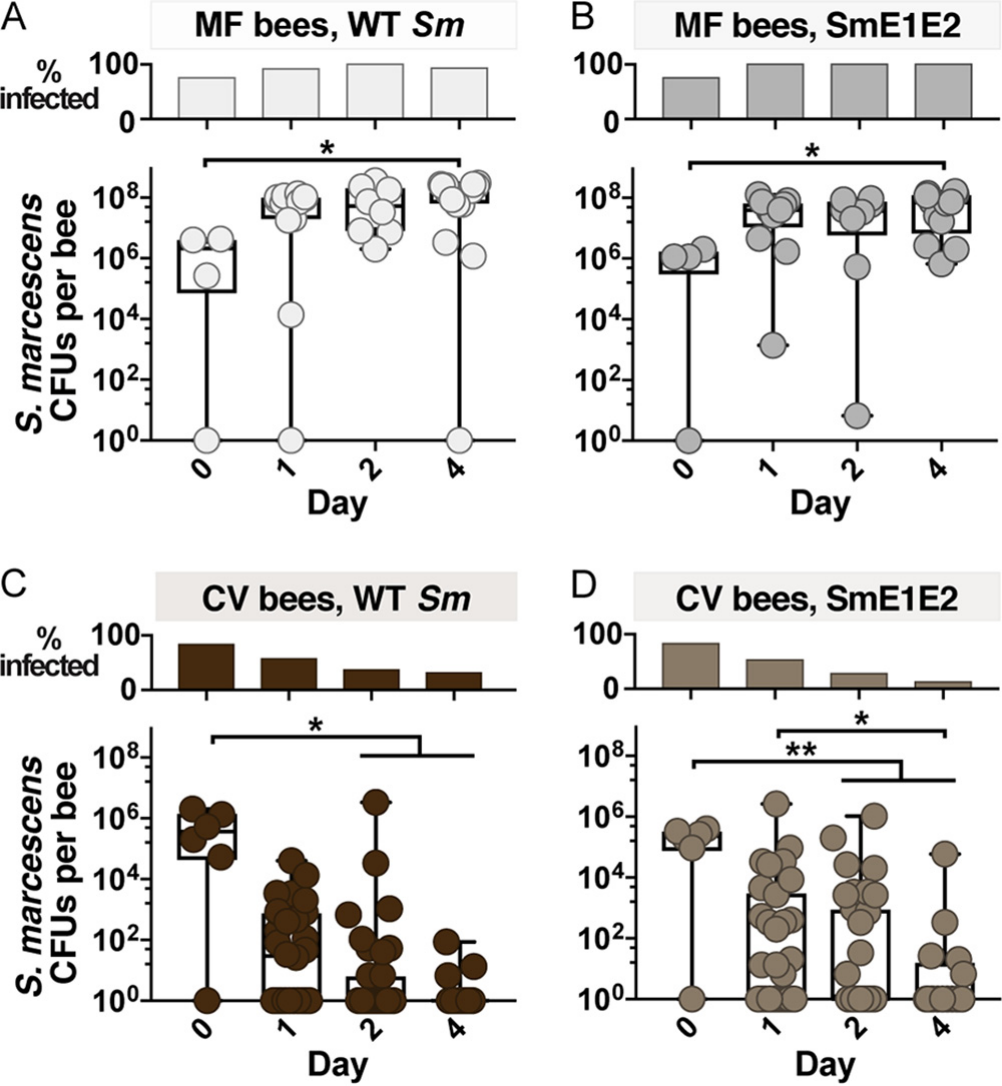
T6SS-deficient Serratia marcescens strains do not differ from the WT strain in fitness within the bee gut. Percentages of sampled bees infected with S. marcescens (top) and abundance of S. marcescens in the midguts and hindguts of individuals (bottom) 1 to 4 days after exposure are shown. MF bees were exposed to WT S. marcescens (A) or to SmE1E2 (B). Conventionalized (CV) bees were exposed to WT S. marcescens (C) or to SmE1E2 (D). Bees were exposed to 4 × 10^8^ S. marcescens cells/ml in sugar syrup for 1 day. Abundance was quantified by counting CFU. Box and whisker plots show range, first and third quartiles, and median. *, *P* < 0.05; **, *P* < 0.005, Kruskal-Wallis test with Dunn’s multiple-comparison test.

To determine whether T6SSs produced by S. marcescens kz11 facilitate colonization, we exposed MF and CV bees to WT or SmE1E2 S. marcescens. In MF bees, WT and SmE1E2 CFU increased after exposure and persisted at high abundance over 4 days (Fig. 4A and B). In contrast, the percentage of CV bees infected and S. marcescens abundance in infected bees decreased over time (Fig. 4C and D). However, we observed no difference in the abundance of WT and SmE1E2 strains, indicating that T6SSs are not required to colonize or persist in MF bees and, further, provide no detectable advantage in CV bees. This result holds true over a range of tested conditions. In a similar experiment, we observed no difference in fitness between WT, SmE1, SmE2, and SmE1E2 strains in MF or CV bees (see Fig. S6). Furthermore, the WT strain does not appear to be more fit than the SmE1E2 strain in monoinoculated bees (see Fig. S7A), and a T6SS-deficient strain (SmC1H1) does not appear to be less fit than the WT strain in CV bees treated with Tet (see Fig. S7B).

## DISCUSSION

Serratia marcescens is a pathogen of many animals, including bees, and is frequently found at low levels in honey bee hives (2, 8, 36). Although deadly when introduced directly into the bee hemolymph, many S. marcescens strains are not lethal when ingested (2). However, S. marcescens has been detected in the bee hemolymph after oral exposure, suggesting that some strains can travel from the gut to the hemolymph, through an unknown route (6). Furthermore, mortality rates are increased in bees whose native gut community has been perturbed by exposure to antibiotics (6, 8) or agrochemicals (7). Therefore, it seems likely that the bee gut microbiota protects hosts by directly antagonizing S. marcescens and/or by reducing access to host tissues.

In this study, S. marcescens kz11, a pathogenic isolate from bees, persisted in guts of MF bees and bees with a perturbed gut community but rapidly declined in guts of CV bees, indicating that the gut microbiota substantially contributes to elimination of this pathogen. Because honey bees do not defecate in captivity (37), reduced abundance is a result of S. marcescens cells dying, rather than leaving the system. We observed elimination of S. marcescens from CV bees within 1 day after exposure in summer (July to September) but only after ≥4 days in fall (October to November), suggesting that seasonal differences in host biology (38) or microbiota composition (39) have some effect. Stimulation of the host immune system by the commensal community may contribute to elimination of S. marcescens. Antimicrobial peptides (AMPs) apidaecin and hymenoptaecin are upregulated in the guts of CV bees or bees monocolonized with S. alvi, relative to MF bees (34, 40), but expression of these AMPs does not differ between CV bees and CV bees exposed to S. marcescens (6), suggesting that this pathway is not part of a host response to S. marcescens infection.

While S. marcescens abundance was reduced in bees colonized by single gut taxa, multiple species were required to attain the level of colonization resistance seen in CV bees, suggesting that bee symbiont species contribute to colonization resistance through different mechanisms. For instance, the microbiota alters bee gut chemistry by reducing oxygen concentration, pH, and redox potential within the gut (13, 41). S. alvi and G. apicola contain T6SSs (19) and may directly antagonize S. marcescens in the ileum. *Lactobacillus* spp. are abundant in the bee rectum (18) and are likely to produce antimicrobial molecules (41–43). Although this trait is not well characterized in bee isolates, *Lactobacillus* species are common probiotics for prevention of Clostridium difficile infections in humans (44). The participation of multiple species in colonization resistance is an important consideration for future investigation of the mechanisms involved. S. alvi appears to compete with S. marcescens for space in the ileum but colonizes only spaces adjacent to the host epithelium (18). Most bee commensals colonize specific regions of the gut (18), which may also explain why monoinoculation does not recapitulate the ability of the natural community to exclude S. marcescens. Intriguingly, CV communities from different bee colonies differed in the level of colonization resistance conferred (see Fig. S2 in the supplemental material), suggesting that the particular combination of species and strains determines the extent of protection, as observed in studies with bumble bees (45–47). Bee guts can also contain yeasts, which are acquired from environmental sources and vary seasonally and between hives (48, 49). These fungi may affect host immune function (50). Serratia marcescens secretes antifungal, as well as antibacterial, T6SS effectors (51) and may interact with yeasts in the bee gut. However, the core gut microbiota, even in the absence of yeasts and low-abundance bacteria, seems to be sufficient to provide colonization resistance.

Because the microbiota provides an obstacle to infection by S. marcescens, we hypothesized that T6SS-mediated antagonism of gut commensals might be important for colonization and pathogenicity, as for some pathogens in mice (30, 32). However, neither T6SS of S. marcescens kz11 contributes to its persistence in the bee gut. The T6SS-1 locus is present in many *Serratia* genomes but did not affect the ability to colonize the gut or to antagonize a range of bacterial competitors under the conditions we tested. In contrast, we observed T6SS-2-mediated killing of G. apicola wkB7, G. apis PEB0162, G. apis PEB0183, E. coli, and other S. marcescens strains *in vitro* but detected no effect on colonization of the gut. The antibacterial properties of T6SS-2 are consistent with previous studies of T6SS structure and function in the related strain S. marcescens Db11 (24). In Db11, the T6SS is constitutively active (52), and the lack of T6SS-mediated killing of bee symbionts by S. marcescens kz11 is unlikely to reflect a lack of T6SS activity. Horizontal transfer of immunity genes, which confer resistance to their cognate toxins, occurs within the gut microbiota of humans (53, 54) and bees (19, 55) and could explain symbiont survival. Alternatively, bee symbionts may be immune to S. marcescens toxins. For example, some toxins secreted by S. marcescens Db11 contain disulfide bonds and depend on a DsbA protein produced by the target cell for proper folding (56). Such toxins are likely to affect other S. marcescens strains but may not be functional within cells of more distantly related organisms. The utility of T6SSs may thus depend on the competitors involved, implying varied and dynamic roles within ecological communities.

## CONCLUSION

Antagonistic interactions between pathogens and commensal microbiota can affect the severity of infection within hosts. We found that the opportunistic pathogen S. marcescens rapidly declines within honey bee guts containing the normal microbiota. Multiple symbiont taxa contribute to colonization resistance, possibly reflecting different niches and mechanisms of inhibition. T6SSs are likely to be important in many bacterial interactions; some pathogens use T6SSs to compete with commensals during infection (30, 32). Serratia marcescens possesses an antibacterial T6SS, but this T6SS does not appear to antagonize the bee gut microbiota. Instead, the S. marcescens T6SSs likely function in competition with close relatives and may have other roles that are still unexplored. These findings are relevant for understanding the ecology of S. marcescens and other opportunistic pathogens, which may often be poorly adapted for competition with host-associated bacteria.

## MATERIALS AND METHODS

Detailed protocols are available in the supplemental material.

### Bacterial strains, plasmids, and culture conditions.

Strains and plasmids used in this study are listed in Table S1 in the supplemental material. E. coli and S. marcescens strains were grown on solid LB plates at 37°C or in liquid LB medium at 37°C at 225 rpm. Bee isolates were grown at 35°C in 5% CO_2_, S. alvi and *Gilliamella* spp. on heart infusion agar supplemented with 5% sheep’s blood (blood HIA) and *Lactobacillus* spp. in MRS broth. When necessary, media were supplemented with antibiotics, i.e., 50 μg/ml kanamycin (Km), 20 μg/ml chloramphenicol (Cm), 60 μg/ml spectinomycin (Sp), 100 μg/ml carbenicillin (Cb), 12.5 μg/ml gentamycin (Gm), 100 μg/ml Rif, 15 μg/ml Tet, or 1 μg/ml anhydrotetracycline (ATc), or with 2,6-diaminopimelic acid (DAP) (0.3 mM).

### Construction of T6SS-deficient mutants.

To create isogenic strains of S. marcescens kz11 with inactive T6SSs (see Table S1), genes encoding required components were replaced with antibiotic resistance markers Km^r^ or Cm^r^. Km^r^ was also inserted downstream of T6SS-1 to generate a Km^r^ strain with a WT phenotype. Plasmids used for allelic exchange contained antibiotic resistance markers flanked by ∼1-kb sequences homologous to regions on either side of the target gene, as well as a Tet-inducible toxin (Tse2) for counterselection. Plasmids were constructed using Golden Gate assembly (see the supplemental material). Plasmids were transformed into the donor strain E. coli MFD*pir* (a DAP auxotroph) through electroporation and then transferred into the recipient strain through conjugation. Transconjugants were selected for and then streaked on LB medium with Tet, ATc, and Km or Cm for selection. PCR was used to verify replacement.

### Competition assays.

Competition assays were used to measure T6SSs-mediated antagonism of E. coli, other S. marcescens strains, or bee gut isolates by S. marcescens kz11. E. coli K-12-Tn*7*-Gm^r^ and S. marcescens WT, SmE1, SmH1, SmE2, SmH2, SmE1E2, and SmH1H2 strains were mixed in a 1:4 ratio, and 25-μl droplets (∼10^6^ E. coli cells) were spotted on LB medium and incubated for 4 h. For all other competition assays, target and attacker were mixed at a 1:10 ratio, and 25-μl droplets (∼10^7^ target cells) were spotted on blood HIA and incubated for 4 h at 35°C in 5% CO_2_. E. coli K-12 was included as a target strain in each set of competition assays, as a control for T6SS activity. Cells from competitions with E. coli and S. marcescens target strains were collected and suspended in 500 μl PBS. Some *Gilliamella* strains were difficult to recover from agar; therefore, agar plugs were excised for competitions with E. coli, S. alvi, and *Gilliamella* target strains, placed in 500 μl PBS, and vortex-mixed to recover the cells. Serial dilutions (1:10) were prepared, and 10 μl of each dilution was spotted in triplicate on selective and nonselective media. Colonies were counted and data visualization and statistical analyses were performed with Prism 7.

### Honey bee experiments.

MF bees were obtained by removing pupae, which naturally lack gut symbionts, from hives maintained by the laboratory, as described previously (57). CV bees and bees with defined communities were obtained by feeding pollen soaked in a mixture of sucrose-PBS (25% sucrose (w/v), 0.5X PBS) and either nurse gut homogenate or 8 × 10^8^ bacterial cells from culture to MF bees within 2 days after emergence. Control MF bees were given pollen soaked in sterile sucrose-PBS. Tet-treated bees were fed 450 μg/ml Tet in sucrose syrup for 4 days, starting 5 days after inoculation. Bees were provided with 1 ml of S. marcescens at an OD600 of 0.5 in 1:1 sucrose or sterile sucrose 5 days after inoculation with gut bacteria or 1 day after the end of Tet treatment. After 1 day, feeding tubes containing S. marcescens were replaced with sterile sucrose. Preliminary experiments indicated that this approach did not increase variability in S. marcescens abundance, relative to hand-feeding a defined number of cells to each bee. To quantify S. marcescens, midguts and hindguts of individual bees were extracted and homogenized in 200 μl PBS. Serial dilutions were prepared, and 10 μl of each dilution was spotted in triplicate on LB agar supplemented with antibiotics. Colonies were counted to estimate S. marcescens CFU per gut.

### Quantification of bee gut symbionts.

DNA was extracted from gut homogenate using the cetyltrimethylammonium bromide (CTAB) method, and quantitative PCR (qPCR) for absolute quantification of *Gilliamella* spp. and S. alvi 16S rRNA gene copies was performed as described by Powell et al. (57), using primers listed in Table S2 in the supplemental material. Quantification was performed in triplicate for each biological replicate.

### FISH microscopy.

Bees were dissected 1 or 3 days after S. marcescens exposure. Guts were fixed in Carnoy’s solution, embedded in paraffin, and cut into 7-μm-thick sections. Fluorescent probes specific to 16S rRNA sequences of S. alvi and S. marcescens (see Table S2) were hybridized to sections, as described previously (18, 58). Sytox blue (50 mM) or green (0.5 mM) dyes were used to label host and microbial DNA. Images were acquired using a Zeiss 710 confocal microscope, except where noted in the figure legends, and processed using ImageJ (59, 60).
