# Supplementary material for: The Gut Microbiota Protects Bees from Invasion by a Bacterial Pathogen
Source: Microbiol Spectr. 2021 Sep 15;9(2):e00394-21. doi: 10.1128/Spectrum.00394-21 (PMC8557934; doi:10.1128/Spectrum.00394-21)
Supplement: SUPPLEMENTAL FILE 1 — Supplemental material. Download SPECTRUM00394-21_Supp_1_seq7.pdf, PDF file, 1.6 MB [file spectrum00394-21_supp_1_seq7.pdf]

Supplemental Material

For: The gut microbiota protects bees from invasion by a bacterial pathogen

Margaret I. Steele, Erick V. S. Motta, Tejashwini Gattu, Daniel Martinez, and Nancy A. Moran

Contents: **Page**

Contents:	Page
Supplementary Methods	2
Figure S1	5
Figure S2	6
Figure S3	7
Figure S4	8
Figure S5	9
Figure S6	10
Figure S7	11
Table S1	12
Table S2	13
Supplementary References	16

Supplementary Methods

Identification of T6SS genes in S. marcescens isolates. Many genes encoding core structural components of T6SSs were identified in *S. marcescens* kz11 by the NCBI Prokaryotic Genome Annotation Pipeline (1, 2). The remaining structural genes were identified based on synteny with annotated T6SS loci from other *S. marcescens* strains. Geneious 10.1.3 (3) was used to visualize T6SS loci in *S. marcescens* kz11. BLAST 2.6.0 (4) was used to search 670 *Serratia* spp. genomes from the NCBI RefSeq database for sequences homologous (>90% nucleotide identity and >70% coverage) to the T6SS loci in kz11.

Construction of plasmids for allelic exchange. Primers and gene blocks used in plasmid construction are described in Table S2. To synthesize an allelic exchange vector with a tetracycline-inducible marker for counterselection (pCS1), XbaI sites were added during PCR amplification of *tse2* and *tetR* genes from pAOJ15 (5) and restriction cloning was used to insert these genes into the suicide vector pBTK599s (6).

Plasmids for allelic exchange were constructed through Golden Gate assembly, as previously described (6, 7). Briefly, ~1kb regions on either side of the target gene were PCR amplified with Phusion polymerase. Golden Gate assembly with NEB BsaI-HFv2 and Promega T4 DNA Ligase was used to assemble PCR products into homology cassettes, with the amplified regions flanking an antibiotic resistance gene (either Km^R from pBTK229 or Cm^R from pYTK001-Cm^R), which were in turn flanked by BsmBI sites from pYTK002 and pYTK072, in a ColE1-Amp^R backbone (pYTK095). To create pYTK001-Cm^R, gene blocks encoding the ColE1 origin of replication from pYTK001 and the Cm^R gene from pYTK001 with promoter and terminator from pBTK229 were combined using Golden Gate assembly. Assembly products were used to transform *E. coli* DH5α, transformants were selected for on LB with antibiotics, and plasmid sequences were verified through diagnostic PCR and Sanger sequencing. A second Golden Gate assembly, using NEB BsmBI and Promega T4 DNA ligase, was used to transfer the homology cassettes from the pYTK095 backbone into pCS1. Golden Gate assembly products were transformed into *E. coli* EC100D*pir*. As before, assemblies were verified by PCR and Sanger sequencing, and plasmids were transformed into the donor strain *E. coli* MFD*pir* through electroporation.

Allelic exchange plasmids were transferred to *S. marcescens* kz11 through conjugation. Donor strains were grown overnight in LB with DAP, Sp, and either Km or Cm. The recipient strain was grown in LB without antibiotics. Donor and recipient cells were washed three times with phosphate buffered saline (PBS), then mixed in a 1:10 ratio and spotted on LB DAP plates. Conjugations were incubated at 37ºC for 6 hours, at which point cells were collected, washed three times with PBS, and spread on LB with Km or Cm. After overnight incubation at 37ºC, colonies were collected and streaked out on LB plates containing Tet, ATc, and either Km or Cm to select for cells in which a second recombination event had excised the plasmid backbone from the bacterial genome. PCR was used to verify replacement of the target gene using one primer that anneals within the antibiotic resistance gene and a second primer that anneals outside of the region of homology used.

Site-specific integration of gentamycin resistance cassette. The mini-Tn7 system was used to insert a gentamycin resistance cassette into the genomes of *E. coli* K12 and *G. apicola* wkB7.

Triparental conjugations were performed as previously described (8) using the pTNS2 helper plasmid and *E. coli* MFD*pir* as the donor strain.

***In vitro* competition assays.** Competitions between WT, SmE1, SmH1, SmE2, SmH2, SmE1E2, and SmH1H2 *S. marcescens* strains and *E. coli* were performed as follows. *S. marcescens* strains were grown overnight on LB plates supplemented with antibiotics, while *E. coli* K12-Tn7-Gm^R was grown overnight in LB broth. Each strain was suspended to an OD of 0.5 in LB, 100µl attacker was mixed with 25µl target, and 25µl of the mixture was spotted on LB in triplicate. Cells were collected after 4 h and suspended in 500µl PBS. 1:10 serial dilutions were prepared and 10µl of each dilution was spotted on LB and LB supplemented with Gm.

To measure intraspecific T6SS-mediated competition under aerobic conditions, *S. marcescens* strains Db11, kz11, and kz19 were domesticated on LB Rif plates. Two colonies were picked and Rif^R mutants were grown overnight at 37ºC. *S. marcescens* kz11 WT, SmE1, SmE2, and SmE1E2 were grown overnight on LB plates supplemented with antibiotics, then suspended in liquid LB. Cells were washed three times in PBS to remove antibiotics. Target and attacker ODs were adjusted to 1 and 10, respectively, and 100µl volumes of target and attacker cells were mixed. 25µl droplets were spotted on LB media. After 6 h, cells were collected from plates and suspended in 500µl PBS. Serial dilutions were prepared and 10µl droplets of each dilution were spotted in triplicate on LB Rif.

To determine the importance of T6SSs for antagonism of bee gut microbes and *S. marcescens* strains under microaerophilic conditions, *G. apicola* wkB1, *G. apicola* PEB0154, *G. apis* PEB0162, and *G. apis* PEB0183 were domesticated on blood HIA Rif. *S. alvi* wkB2-mariner-Gm^R, *G. apicola* wkB7-Tn7-Gm^R, *G. apicola* wkB1-Rif$^{\underline{R}}$, *G. apicola* PEB0154-Rif^R, *G. apis* PEB0162-Rif^R, and *G. apis* PEB0183-Rif^R were grown on blood HIA for 2 d, *S. marcescens* strains WT, SmE1E2, Db11-Rif^R, kz19-Rif^R, and kz11-Rif^R were grown overnight on LB agar, and *E. coli* K12-Tn7-Gm^R was grown overnight in LB broth. WT and SmE1E2 *S. marcescens* colonies were scraped from plates, suspended in PBS, and then diluted to an OD of 1. Target strains (*S. alvi*, *G. apicola*, *G. apis*, and other *S. marcescens* strains) were suspended in PBS and diluted to an OD of 0.1. 100µl of each target strain was mixed with 100µl of each attacker and 25µl of the mixture was spotted onto blood HIA and incubated at 35ºC in 5% CO_2. After 4 h, cells were collected from plates, placed in tubes with 500µl PBS, and vortexed to suspend cells. Cells from intraspecific competition assays were collected using an inoculation loop, while cells from *G. apicola*, *G. apis*, and *S. alvi* competitions were collected by excising the agar around the cells and vortexing it in PBS. Competitions with *E. coli* as the target strain were included in both treatments as a positive control for T6SS activity. Serial dilutions were prepared and then spotted in triplicate on selective and non-selective blood HIA and LB plates to enumerate both target and attacker CFUs. Data visualization and statistical analyses were performed with Prism 7.

Honey bee colonization assays.

MF bees and experimental inoculation with gut symbionts: Microbiota-free (MF) bees were reared, as previously (9), by removing pupae, which naturally lack gut symbionts, from hives maintained by the lab. Bees were kept in sterile containers and supplied with filter-sterilized 1:1 sucrose solution and gamma-irradiated sterile pollen. Conventionalized and mono-inoculated

bees were obtained by exposing MF bees to gut bacteria added to their pollen supply within 48 hours of emergence. To inoculate conventionalized bees, guts of nurse bees from the original hive were collected and homogenized in 600µl sucrose-PBS (25% sucrose, 50% PBS), which was then used to soak sterile pollen. To colonize bees with single strains or defined communities, bacterial cultures were grown on blood HIA for 2 d, then collected and diluted to $8x10^8$ cells ml^{-1} in sucrose-PBS, and mixed with sterile pollen. Bees in MF treatment groups were provided with pollen soaked in sterile sucrose-PBS. Gut homogenate for inoculating CV bees was obtained from a nurse bee from the same hive as the bees to be inoculated, except for the experiment shown in Fig S2, in which bees from one hive were exposed to gut homogenate from nurse bees collected from one of two different hives.

Tetracycline treatment: Tetracycline-treated (Tet) bees were fed 450µg ml^{-1} Tet in sucrose syrup for 4 d, starting 5 d after inoculation. In small-scale preliminary experiments, Tet bees were similar to MF bees. In large-scale experiments, Tet bees were much more variable, suggesting that the number of bees per cage during treatment may affect the biological variation observed in the effect of tetracycline on the microbiota.

S. marcescens exposure: *S. marcescens* strains were grown overnight on selective media, collected into sterile PBS, and suspended to an OD of 0.5 in 1:1 sucrose. Bees were immobilized by chilling and then split between sterile cages. Each cage was provided with 1ml *S. marcescens* in sucrose, while control cages were provided with sterile sucrose. After 1 d, bees were immobilized and feeding tubes containing sucrose with *S. marcescens* were replaced with fresh feeding tubes containing only sterile sucrose. In some experiments, bees were sampled at this time (immediately after exposure). Bees were then sampled at 1 d intervals, and sucrose solution was replenished as necessary. To quantify *S. marcescens* abundance, midguts and hindguts were dissected out of immobilized bees and homogenized in 200µl sterile PBS. 1:10 serial dilutions were prepared in PBS, and 10µl of each dilution was spotted in triplicate on LB supplemented with antibiotics. The resulting distribution of colonies was used to estimate the number of *S. marcescens* CFUs in the gut.

Fluorescence *in situ* Hybridization (FISH) microscopy. MF bees were inoculated with *S. alvi* wkB2 or nurse gut homogenate (CV). After 5 d, bees were exposed to OD 0.5 WT or SmE1E2 *S. marcescens* in sucrose solution for 1 d. Bees were dissected 1 and 3 d after the end of exposure and guts were stored in 100% ethanol at -20ºC. Preserved ileums were fixed in Carnoy's solution (60% ethanol, 30% chloroform, 10% glacial acetic acid) overnight at room temperature, washed three times in absolute ethanol, then xylene. Ileums were embedded in Tissue Prep paraffin formulation (FisherScientific) and cut into 7µm-thick sections using a Leica RM2245 rotary microtome. Sections were mounted on FisherBrand SelectFrost Adhesion microscope slides, which were then washed three times in xylene to remove paraffin, rinsed with absolute ethanol, then molecular grade water. Fluorescent probes specific to 16S rRNA sequences of *S. alvi* and *S. marcescens* (SI Table S2) were hybridized to sections overnight (20mM Tris-HCl, 0.9M NaCl, 0.01% SDS, 30% Formamide, 100µM each probe), as described previously (10). Sytox blue (50mM) or green (0.5mM) dyes were included in the hybridization buffer to label host and microbial DNA. Slides were washed in PBSTx (1X PBS, 0.3% Triton-X), SlowFade reagent (Invitrogen) was added, and a coverslip attached. Images were acquired using a Zeiss 710 confocal microscope (Fig. 2) or Nikon inverted fluorescence microscope (Fig. S3) and processed using ImageJ (11, 12).

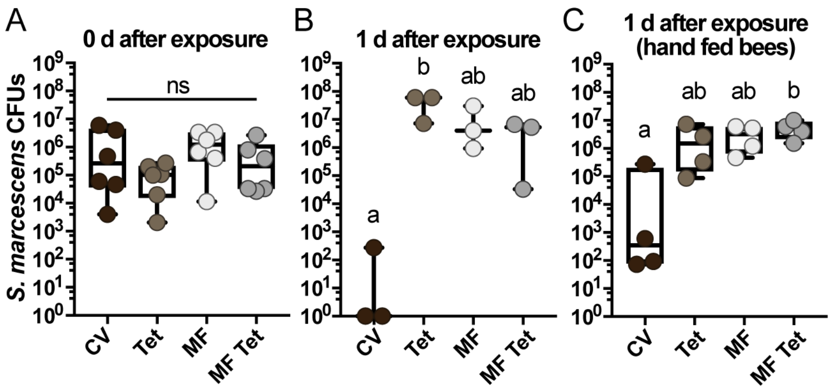

Figure S1. *S. marcescens* abundance in guts of bees exposed to 4x10^8 *S. marcescens* cells ml^{-1} in sugar syrup for 1 d (AB) or hand-fed approximately 4x10^6 cells (C). Individual bees were sampled immediately (A) or 1 d after exposure (BC). Abundance was quantified by counting CFUs. Box and whisker plot show the minimum, first quartile, median, third quartile, and maximum. Letters indicate significant differences between treatment groups. Kruskal-Wallis test with Dunn's multiple comparisons test, $p < 0.05$.

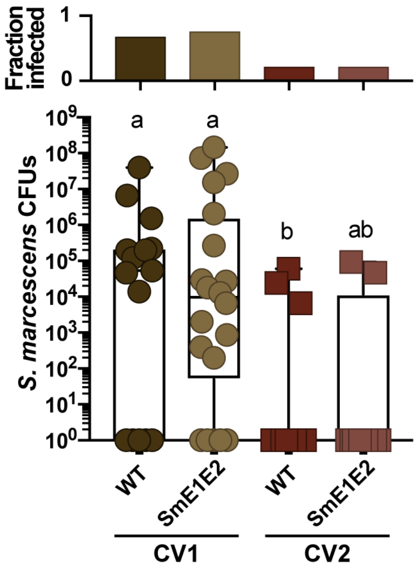

Figure S2. *S. marcescens* abundance in guts of bees colonized by different "conventional" communities. Age-controlled, microbiota-free honey bees from a single hive were inoculated with gut homogenate from a nurse bee from two different hives (CV1 and CV2). After 5 d, bees were exposed to WT or SmE1E2 *S. marcescens*. The fraction of bees infected (upper panel) and the abundance of *S. marcescens* in the midgut and hindgut (lower panel) were measured 10 d after exposure. Letters indicate significant differences between treatment groups. Kruskal-Wallis test with Dunn's multiple comparisons test, $p < 0.05$.

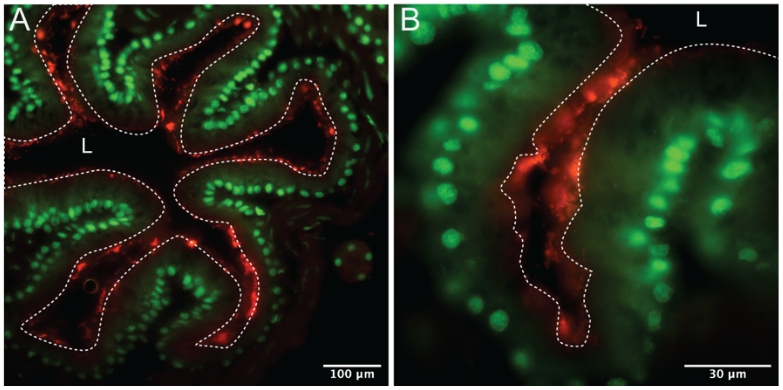

Figure S3. In MF bees, *S. marcescens* (red) colonizes spaces usually occupied by members of the commensal microbiota. *S. marcescens* was visualized in ileum cross-sections using a fluorescent probe that hybridizes specifically to its 16S rRNA. Sytox green was used to stain host nuclei (green). Dashed lines mark the interior surface of the host epithelium. L, lumen of gut. Images were obtained using a Nikon inverted fluorescence microscope.

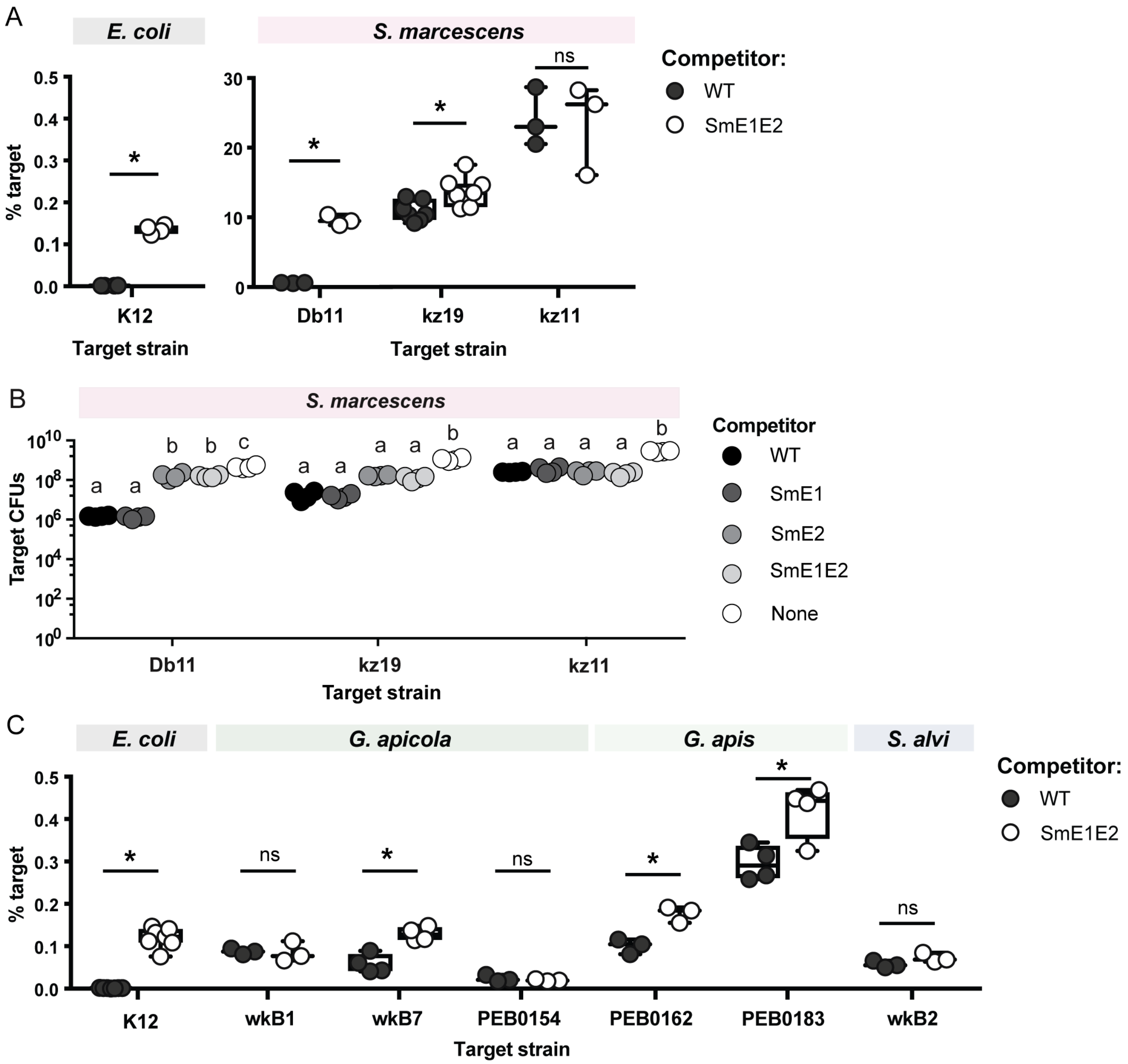

Figure S4. (A) The percent of recovered cells (target CFUs/total CFUs x 100) that were *E. coli* K12 or *S. marcescens* Db11, kz19, or kz11 after 4 h coculture with WT *S. marcescens* kz11 (black) or SmE1E2 (white) in microaerophilic conditions. Cocultures were initially 10% target. Unpaired t-test; *, $p < 0.05$ (B) *S. marcescens* Db11, kz19, and kz11 CFUs after 6 h coculture with WT, SmE1, SmE2, SmE1E2 or buffer in aerobic conditions. One-way ANOVA with Holm-Sidak's multiple comparisons test. Letters indicate significant differences between groups, $p < 0.05$. (C) Recovery of *E. coli* K12 and gut commensals *G. apicola* wkB1, wkB7, and PEB0154; *G. apis* PEB0162 and PEB0183; and *S. alvi* wkB2 after 4 h coculture with WT or SmE1E2 *S. marcescens* in microaerophilic conditions, 1:10 starting ratio. Letters indicate significant differences between treatment groups. Unpaired t-test; *, $p < 0.05$; ns, no significant difference. Target CFUs were measured through plate counts on selective media.

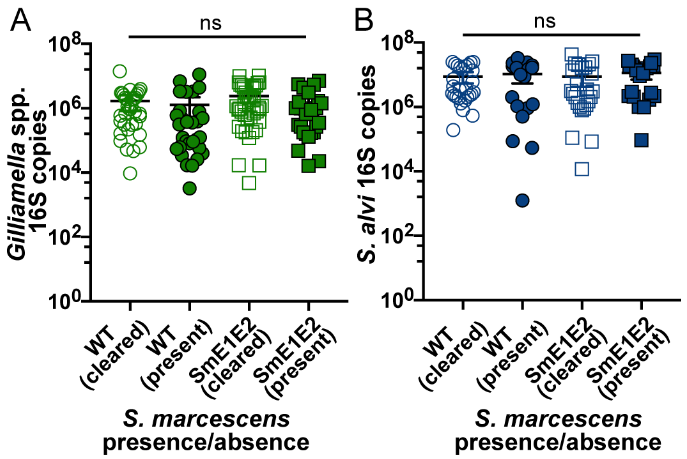

Figure S5. (A) *Gilliamella* spp. and (B) *S. alvi* 16S rRNA gene copies within guts of CV bees infected with WT or SmE1E2 *S. marcescens*. All bees were initially infected with *S. marcescens*. White circles and squares, no *S. marcescens* CFUs detected at sampling time. Filled circles and squares, *S. marcescens* CFUs present. Ordinary one-way ANOVA – no significant difference between means.

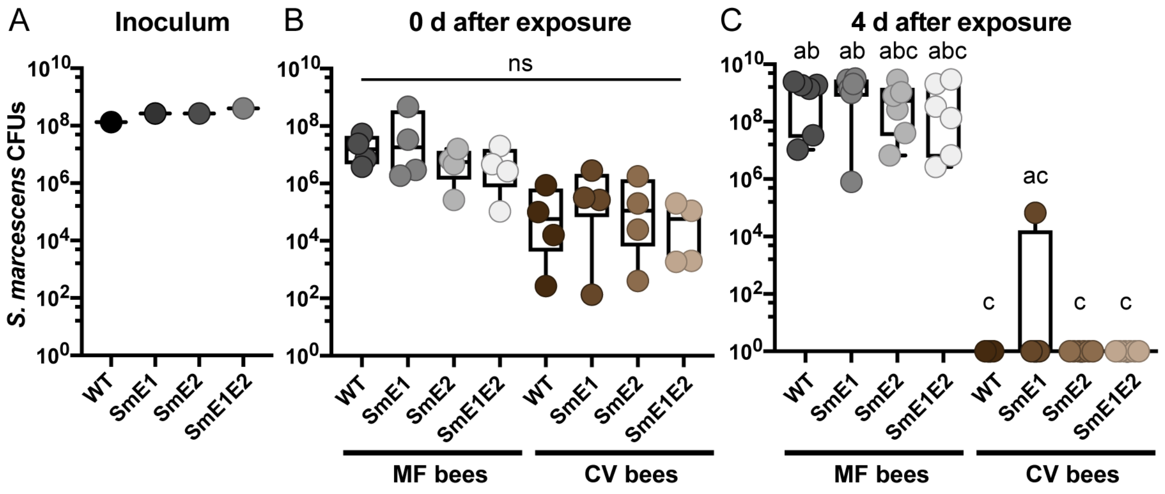

Figure S6. Abundance of WT *S. marcescens* or T6SS-deficient mutants SmE1, SmE2, or SmE1E2 in (A) sucrose syrup fed to bees, (B) microbiota-free (MF) and conventionalized (CV) bees immediately after exposure, and (C) MF and CV bees 4 d after exposure. CFUs were quantified by counting colonies. Box and whisker plot show range, first and third quartiles, and median. Kruskal-Wallis test with Dunn’s multiple comparisons test, different letters indicate significant differences between medians, $p < 0.05$.

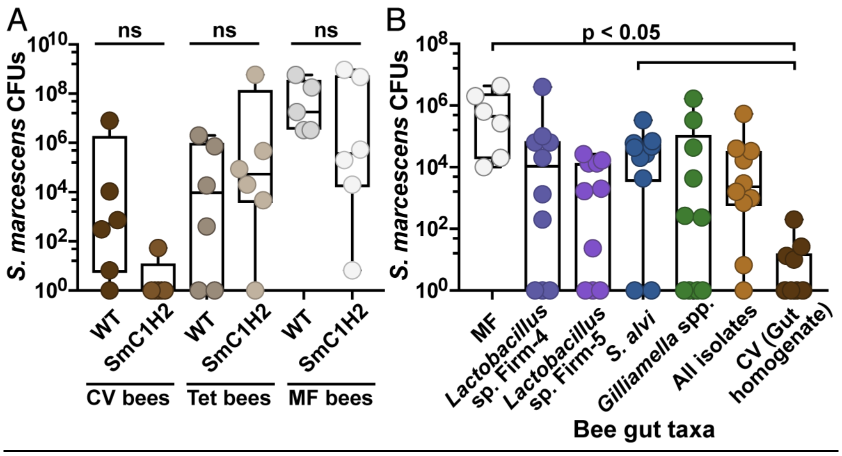

Figure S7. (A) Total abundance of *S. marcescens* mutants in the midgut and hindgut of CV bees, CV bees treated with tetracycline (Tet), or microbiota-free bees (MF). Bees were hand fed approximately 4×10^6 cells of WT *S. marcescens* or *S. marcescens ΔtssC1ΔtssH2*. (B) *S. marcescens* SmE1E2 abundance in bees colonized with core gut taxa. Bees were exposed to 4×10^8 *S. marcescens* SmE1E2 cells ml^{-1} in sugar syrup 5 d after inoculation with symbionts (*Lactobacillus* sp. Firm-4 DSM 26254, DSM 26255; *Lactobacillus* sp. Firm-5 wkB8, wkB10; *S. alvi* wkB2; *G. apicola* wkB1, PEB0154 and *G. apis* PEB0162, PEB0183; all isolates; or homogenized gut of a hive bee). Abundance was quantified 1 d after exposure by counting CFUs per bee. Box and whisker plot show range, first and third quartiles, and median. Kruskal-Wallis test with Dunn's multiple comparisons test.

Table S1. Bacterial strains and plasmids used in this study.

**Strains**			
Escherichia coli	DH5α	Cloning host	
	EC100D*pir*	Cloning host for plasmids with R6K origin	
	MFD*pir*	Donor strain for conjugations	(13)
	K12	Wild-type strain	
	K12-Tn7-Gm^R	Target strain for competition assays	(this study)
Snodgrassella alvi	wkB2	Wild-type strain	
	wkB2-mariner-Gm^R	Wild-type strain, neutral insertion of mariner transposon carrying Gm^R	(14)
Gilliamella apicola	wkB1	Wild-type strain	
	wkB7-Tn7-Gm^R	Wild-type strain, site-specific insertion of Tn7 transposon carrying Gm^R	(this study)
	PEB0154	Wild-type strain	
Gilliamella apis	PEB0162	Wild-type strain	
	PEB0183	Wild-type strain	
Lactobacillus Firm-4	DSM 26254	Wild-type strain	
	DSM 26255	Wild-type strain	
Lactobacillus Firm-5	wkB8	Wild-type strain	
	wkB10	Wild-type strain	
Serratia marcescens	kz11	Wild-type strain, prodigisin producing strain	
	WT	Insertion of Km^R outside of T6SS loci	(this study)
	SmE1	*ΔtssE1*, Km^R	(this study)
	SmH1	*ΔtssH1*, Km^R	(this study)
	SmE2	*ΔtssE2* , Cm^R	(this study)
	SmH2	*ΔtssH2*, Cm^R	(this study)
	SmC1	*ΔtssC1*, Km^R	(this study)
	SmE1SmE2	*ΔtssE1ΔtssE2*, Km^R, Cm^R	(this study)
	SmH1SmH2	*ΔtssH1ΔtssH2* Km^R, Cm^R	(this study)
	Db11	Wild-type strain, spontaneous Rif^R mutant	
	kz19	Wild-type strain, spontaneous Rif^R mutant	

**Plasmids**		
pUC18T-mini-Tn7T-Gm	mini-Tn7 transposon carrying Gm^R	(8)
pTNS2	Tn7 transposase	(8)
pYTK001	Golden gate part plasmid, entry vector	(7)
pBTK229	Golden gate part plasmid, Km^R, type 3 part	(6)
PYTK001-Cm^R	Golden gate part plasmid, Cm^R, type 3 part plasmid	(this study)
pYTK002	Golden gate part plasmid, type 1 part	(7)
pYTK072	Golden gate part plasmid, type 5 part	(7)
pYTK095	Golden gate part plasmid, type 6-8 part	(7)
pBTK599s	Suicide vector with origin of transfer, GFP, Sp^R	(6)
pAOJ15	Backbone for counter selectable plasmids, tetracycline-inducible *tse2* toxin gene	(5)
pCS1	*tetR-tse2* from pAOJ15 cloned into pBTK599s backbone	(this study)

Table S2. Primers, gene blocks, and probes used in this study.

Template/target	Purpose	Name	Sequence	Source
S. marcescens kz11	PCR: Neutral insertion, Golden Gate type 2 part (BsaI sites)	MS6Sm80	GCATCGTCTCATCGGTCTCAAACGTCTAGAGTCCTTCTCCATGTTCATCA	
		MS6Sm81	ATGCCGTCTCAGGTCTCACATACAAGCTGGGTATCACTATGA	
S. marcescens kz11	PCR: Neutral insertion, Golden Gate type 4 part (BsaI sites)	MS6Sm82	GCATCGTCTCATCGGTCTCAATCCAGTGATACCCAGCTTGTTTT	
		MS6Sm83	ATGCCGTCTCAGGTCTCACAGCTCTAGAAGAAATGCCTGGTAGGAGTA	
S. marcescens kz11	PCR: ΔtssE1, Golden Gate type 2 part (BsaI sites)	MS6Sm60	GCATCGTCTCATCGGTCTCAAACGCATTACCAGTTGGAAGGCATG	
		MS6Sm61	ATGCCGTCTCAGGTCTCACATAGCTTTGATGTCTCACGCTGAG	
S. marcescens kz11	PCR: ΔtssE1, Golden Gate type 4 part (BsaI sites)	MS6Sm62	GCATCGTCTCATCGGTCTCAATCCTTAACGCTGGCGAGACATATACA	
		MS6Sm63	ATGCCGTCTCAGGTCTCACAGCTCTAGAGCGCGAACAAGATCTCGAAC	
S. marcescens kz11	PCR: ΔtssH1, Golden Gate type 2 part (BsaI sites)	MS6Sm54	GCATCGTCTCATCGGTCTCAAACGAGCTCATCACCTCTATCGTGCAGCG	
		MS6Sm55	ATGCCGTCTCAGGTCTCACATACATCAGCAGCCCTCTTTCCA	
S. marcescens kz11	PCR: ΔtssH1, Golden Gate type 4 part (BsaI sites)	MS6Sm56	GCATCGTCTCATCGGTCTCAATCCCATACCGTTTCCACCCTGAA	
		MS6Sm57	ATGCCGTCTCAGGTCTCACAGCTCTAGAAAACCGTAGCGCGAGAAGA	
S. marcescens kz11	PCR: ΔtssE2, Golden Gate type 2 part (BsaI sites)	MS6Sm73	GCATCGTCTCATCGGTCTCAAACGTCTAGAATATTGTCGACCACCAGATG	
		MS6Sm75	ATGCCGTCTCAGGTCTCACATATATCCGCTGGAGTTTCTGTTC	
S. marcescens kz11	PCR: ΔtssE2, Golden Gate type 4 part (BsaI sites)	MS6Sm77	GCATCGTCTCATCGGTCTCAATCCGTCCTGACGATAGCGATAGC	
		MS6Sm79	ATGCCGTCTCAGGTCTCACAGCTCTAGATGACTTTCAAACTGGTGGAA	
S. marcescens kz11	PCR: ΔtssH2, Golden Gate type 2 part (BsaI sites)	MS6Sm66	GCATCGTCTCATCGGTCTCAAACGTCTAGACAGATAACCTTCCTGATGGATG	
		MS6Sm67	ATGCCGTCTCAGGTCTCACATACATATCGATTTCCGCAACAC	
S. marcescens kz11	PCR: ΔtssH2, Golden Gate type 4 part (BsaI sites)	MS6Sm70	GCATCGTCTCATCGGTCTCAATCCTTTCAATGAGCCATAGACCC	
		MS6Sm71	ATGCCGTCTCAGGTCTCACAGCTCTAGAGTTTCGATCTTCAGTTTCGG	
S. marcescens kz11	PCR: ΔtssC1, Golden Gate type 2 part (BsaI sites)	MS6Sm84_2F	GCATCGTCTCATCGGTCTCAAACGATGTCATCTTGCTGTTGGAT	
		MS6Sm85_2R	ATGCCGTCTCAGGTCTCACATAAATGGTCAATACGACATCCT	
S. marcescens kz11	PCR: ΔtssC1, Golden Gate type 4 part (BsaI sites)	MS6Sm86_4F	GCATCGTCTCATCGGTCTCAATCCGAGGATGTGGAAGATGATCC	
		MS6Sm87_4R	ATGCCGTCTCAGGTCTCACAGCCCTGCAATTAGTGTTGTGAC	
pAOJ15	PCR: *tetR-tse2* (XbaI sites)	MSCS03	ATATCCTCTAGAATGTTGTCTACATGGCTCTG	
		MSCS04	ATATCCTCTAGATTCAGGTTCATGGTTCACTC	
pYTK001-derived plasmids	PCR: Verification of Golden Gate assembly	MSGG07	CTGATTCTGTGGATAACCGT	
		MSGG08	ATTTGATATCGAGCTCGCTT	
pYTK095-derived plasmids	PCR: Verification of Golden Gate assembly	GG-stage1-FW	TCACAGACATTAACCCACAG	
		GG-stage1-RV	TCTTGCTTAGTTGTGAGTCG	
pBTK599s-derived plasmids	PCR: Verification of Golden Gate assembly	BTK599s_FW2	GAACCGAACAGGCTTATGT	
		BTK599s_RV2	TCCTTCTTCACTGTCCCTTA	
KmR	PCR: Verification of allelic exchange	MSAEV02	CCTTATTTTTGACGAGGGGA	
		MSAEV04	CCCGTTGAATATGGCTCATA	
Neutral insertion	PCR: Verification of allelic exchange	MSAEV27	ATTCAGCAAAGCCAGAAGAA	
		MSAEV28	TCCAAGAGCGCAATATATCC	

Template/target	Purpose	Name	Sequence	Source
SmE1	PCR: Verification of allelic exchange	MSAEV11<br>MSAEV12	CTTTTATCGGTTCTTGCACC<br>GATGGCGATGAAGGTATCTT	
SmE2	PCR: Verification of allelic exchange	MSAEV31<br>MSAEV32	GAGGTATTTCAGATCGCTGT<br>GTGAAGCGATCGTTGAAATG	
SmH1	PCR: Verification of allelic exchange	MSAEV09<br>MSAEV10	GAAGATACCTTCATCGCCAT<br>TAACCGAAATCCAGATCGTC	
SmH2	PCR: Verification of allelic exchange	MSAEV29<br>MSAEV30	TTTGAGCATGATCTCCGTTT<br>AAACACGATCTGTTTCAGCT	
SmC1	PCR: Verification of allelic exchange	MSAEV40<br>MSAEV41	CGCGACTATTTGAAACAGTT<br>ACGCTGAGCTTATCGAATAG	
Gene block	CmR (BsmBI sites)	CmR part3	GCATCGTCTCATCGGTCTCATATGCTGATCCTTCAACTCAG<br>CAAAAGTTCGATTTATTCAACAAAGCCACGTTGTGTCTCAA<br>AATCTCTGATGTTACATTGCACAAGATAAAAATATATCATC<br>ATGAACAATAAAACTGTCTGCTTACATAAACAGTAATACAA<br>GGGGTGTTATGGAGAAAAAAATCACTGGATATACCACCGT<br>TGATATATCCCAATGGCATCGTAAAGAACATTTTGAGGCAT<br>TTCAGTCAGTTGCTCAATGTACCTATAACCAGACCGTTCAG<br>CTGGATATTACGGCCTTTTTAAAGACCGTAAAGAAAAATAA<br>GCACAAGTTTTATCCGGCCTTTATTCACATTCTTGCCCGCCT<br>GATGAATGCTCATCCGGAATTTCGTATGGCAATGAAAGAC<br>GGTGAGCTGGTGATATGGGATAGTGTTCACCCTTGTTACA<br>CCGTTTTCCATGAGCAAACTGAAACGTTTTCATCGCTCTGG<br>AGTGAATACCACGACGATTTCCGGCAGTTTCTACACATATA<br>TTCGCAAGATGTGGCGTGTTACGGTGAAAACCTGGCCTAT<br>TTCCCTAAAGGGTTTATTGAGAATATGTTTTTCGTTTCAGCC<br>AATCCCTGGGTGAGTTTCACCAGTTTTGATTTAAACGTGGC<br>CAATATGGACAACTTCTTCGCCCCCGTTTTCACCATGGGCA<br>AATATTATACGCAAGGCGACAAGGTGCTGATGCCGCTGGC<br>GATTCAGGTTCATCATGCCGTTTGTGATGGCTTCCATGTCG<br>GCAGAATGCTTAATGAATTACAACAGTACTGCGATGAGTG<br>GCAGGGCGGGGCGTAATCAGAATTGGTTAATTGGTTGTAA<br>CACGCGGCCGCTCGGTATAATCCTGAGACCTGAGACGGCA<br>T	
Gene block	ColE1 origin of replication (BsmBI sites)	ColE1-BsmBI	ATACGTCTCTGACCAGACCGGTGAAAGTTGGAACCTCTTAC<br>GTGCCCGATCAATCATGACCAAAATCCCTTAACGTGAGTTT<br>TCGTTCCACTGAGCGTCAGACCCCGTAGAAAAGATCAAAG<br>GATCTTCTTGAGATCCTTTTTTTCTGCGCGTAATCTGCTGCT<br>TGCAAACAAAAAAACCACCGCTACCAGCGGTGGTTTGTTT<br>GCCGGATCAAGAGCTACCAACTCTTTTTCCGAAGGTAACTG<br>GCTTCAGCAGAGCGCAGATACCAAATACTGTTCTTCTAGTG<br>TAGCCGTAGTTAGGCCACCACTTCAAGAACTCTGTAGCACC<br>GCCTACATACCTCGCTCTGCTAATCCTGTTACCAGTGGCTG<br>CTGCCAGTGGCGATAAGTCGTGTCTTACCGGGTTGGACTC<br>AAGACGATAGTTACCGGATAAGGCGCAGCGGTCGGGCTG<br>AACGGGGGGTTCGTGCACACAGCCCAGCTTGGAGCGAAC<br>GACCTACACCGAACTGAGATACCTACAGCGTGAGCTATGA<br>GAAAGCGCCACGCTTCCCGAAGGGAGAAAGGCGGACAGG<br>TATCCGGTAAGCGGCAGGGTCGGAACAGGAGAGCGCACG<br>AGGGAGCTTCCAGGGGGAAACGCCTGGTATCTTTATAGTC<br>CTGTCGGGTTTCGCCACCTCTGACTTGAGCGTCGATTTTTG<br>TGATGCTCGTCAGGGGGGCGGAGCCTATGGAAAAACGCC<br>AGCAACGCGGCCTTTTTACGGTTCCTGGCCTTTTGCTGGCC<br>TTTTGCTCACATGTTCTTTCCTGCGTTATCCCCTGATTCTGT<br>GGATAACCGTAGTCGGCGAGACGGAAAGTGAAACT	
S. alvi	Quantitative PCR, 16S rRNA gene	Salv-16S-qtF<br>Salv-16S-qtR	CTTAGAGATAGGAGAGTGCCTT<br>AACTTAATGATGGCAACTAATGACAA	(15)<br>(15)
Gilliamella spp.	Quantitative PCR, 16S rRNA gene	Gamma1-459-qtF<br>Gamma1-648-qtR	GTATCTAATAGGTGCATCAATT<br>TCCTCTACAATACTCTAGTT	(16)<br>(16)
S. alvi	Fluorescence *in situ* hybridization, 16S rRNA	beta-572-AlexaFluor 647	TTAACCGTCTGCGCTCGCTT	(16)
S. marcescens	Fluorescence *in situ* hybridization, 16S rRNA	sm420-AlexaFluor594	CCACCTTCCTCCTCGCT	(this study)

Supplementary References

1. Tatusova T, DiCuccio M, Badretdin A, Chetvernin V, Nawrocki EP, Zaslavsky L, Lomsadze A, Pruitt KD, Borodovsky M, Ostell J. 2016. NCBI prokaryotic genome annotation pipeline. Nucleic Acids Res 44:6614–6624.

2. Haft DH, DiCuccio M, Badretdin A, Brover V, Chetvernin V, O'Neill K, Li W, Chitsaz F, Derbyshire MK, Gonzales NR, Gwadz M, Lu F, Marchler GH, Song JS, Thanki N, Yamashita RA, Zheng C, Thibaud-Nissen F, Geer LY, Marchler-Bauer A, Pruitt KD. 2018. RefSeq: an update on prokaryotic genome annotation and curation. Nucleic Acids Res 46:D851–D860.

3. Kearse M, Moir R, Wilson A, Stones-Havas S, Cheung M, Sturrock S, Buxton S, Cooper A, Markowitz S, Duran C, Thierer T, Ashton B, Meintjes P, Drummond A. 2012. Geneious Basic: An integrated and extendable desktop software platform for the organization and analysis of sequence data. Bioinformatics 28:1647–1649.

4. Camacho C, Coulouris G, Avagyan V, Ma N, Papadopoulos J, Bealer K, Madden TL. 2009. BLAST+: architecture and applications. BMC Bioinformatics 10:421.

5. Armbruster CE, Forsyth-DeOrnellas V, Johnson AO, Smith SN, Zhao L, Wu W, Mobley HLT. 2017. Genome-wide transposon mutagenesis of *Proteus mirabilis*: Essential genes, fitness factors for catheter-associated urinary tract infection, and the impact of polymicrobial infection on fitness requirements. PLoS Pathog 13.

6. Leonard SP, Perutka J, Powell JE, Geng P, Richhart D, Byrom M, Kar S, Davies BW, Ellington AD, Moran N, Barrick JE. 2018. Genetic engineering of bee gut microbiome bacteria with a toolkit for modular assembly of broad-host-range plasmids. ACS Synth Biol https://doi.org/10.1021/acssynbio.7b00399.

7. Lee ME, DeLoache WC, Cervantes B, Dueber JE. 2015. A highly characterized yeast toolkit for modular, multipart assembly. ACS Synthetic Biology 4:975–986.

8. Choi K-H, Schweizer HP. 2006. mini-Tn*7* insertion in bacteria with single *att*Tn*7* sites: example *Pseudomonas aeruginosa*. Nature Protocols 1:153–161.

9. Powell JE, Martinson VG, Urban-Mead K, Moran NA. 2014. Routes of acquisition of the gut microbiota of the honey bee *Apis mellifera*. Appl Environ Microbiol 80:7378–7387.

10. Engel P, James, Rosalind R., Koga R, Kwong WK, McFrederick QS, Moran NA. 2013. Standard methods for research on *Apis mellifera* gut symbionts. Journal of Apicultural Research 52.

11. Rueden CT, Schindelin J, Hiner MC, DeZonia BE, Walter AE, Arena ET, Eliceiri KW. 2017. ImageJ2: ImageJ for the next generation of scientific image data. BMC Bioinformatics 18:529.

12. Schindelin J, Arganda-Carreras I, Frise E, Kaynig V, Longair M, Pietzsch T, Preibisch S, Rueden C, Saalfeld S, Schmid B, Tinevez J-Y, White DJ, Hartenstein V, Eliceiri K, Tomancak P, Cardona

A. 2012. Fiji: an open-source platform for biological-image analysis. Nat Methods 9:676–682.

13. Ferrières L, Hémery G, Nham T, Guérout A-M, Mazel D, Beloin C, Ghigo J-M. 2010. Silent mischief: bacteriophage Mu insertions contaminate products of *Escherichia coli* random mutagenesis performed using suicidal transposon delivery plasmids mobilized by broad-host-range RP4 conjugative machinery. Journal of Bacteriology 192:6418–6427.

14. Powell JE, Leonard SP, Kwong WK, Engel P, Moran NA. 2016. Genome-wide screen identifies host colonization determinants in a bacterial gut symbiont. PNAS 113:13887–13892.

15. Kešnerová L, Mars RAT, Ellegaard KM, Troilo M, Sauer U, Engel P. 2017. Disentangling metabolic functions of bacteria in the honey bee gut. PLOS Biology 15:e2003467.

16. Martinson VG, Moy J, Moran NA. 2012. Establishment of characteristic gut bacteria during development of the honeybee worker. Appl Environ Microbiol 78:2830–2840.
